# Structural equation modeling of the effects of psychological distress and a fear of coronavirus disease 2019 on diabetes care in Japan: a cross-sectional study

**DOI:** 10.1038/s41598-022-20716-4

**Published:** 2022-09-27

**Authors:** Akira Minoura, Takehiro Sugiyama, Teruhide Koyama, Takashi Yoshioka, Takahiro Tabuchi

**Affiliations:** 1 Department of Hygiene, Public Health and Preventive Medicine, Showa University School of Medicine, 1-5-8 Hatanodai, Shinagawa-ku, Tokyo, 142-8555 Japan; 2 Department of Health Services Research, Faculty of Medicine, University of Tsukuba, Ibaraki, 305-8575 Japan; 3 Institute for Global Health Policy Research, Bureau of International Health Cooperation, National Center for Global Health and Medicine, Tokyo, 162-8655 Japan; 4 Diabetes and Metabolism Information Center, Research Institute, National Center for Global Health and Medicine, Tokyo, 162-8655 Japan; 5 Department of Epidemiology for Community Health and Medicine, Kyoto Prefectural University of Medicine, Kyoto, Japan; 6 Department of Preventive Medicine and Public Health, School of Medicine, Keio University, Tokyo, Japan; 7 Center for Innovative Research for Communities and Clinical Excellence (CiRC2LE), Fukushima Medical University, Fukushima City, Fukushima Japan; 8 Center for Cancer Control and Statistics, Osaka Medical Cancer and Cardiovascular Diseases, Osaka, Japan

**Keywords:** Health care, Risk factors

## Abstract

This study aimed to examine the effects of psychological distress and a fear of coronavirus disease 2019 (COVID-19) on diabetes care in Japan. We used data from a 2020 nationwide Internet survey in Japan involving 28,000 respondents aged 15–79 years. The question items included psychological factors (Kessler psychological distress scale and fear of COVID-19), employment, trust in neighbors, informal caregiving, and history of diabetes care. After excluding respondents with comorbidities and those who had not visited the hospital, 625 patients with diabetes were analyzed. Statistical mediation was then examined through a path analysis using structural equation modeling (SEM). Discontinued diabetes care was independently associated with psychological distress (risk ratio = 1.44, 95% confidence interval [1.01–2.06]) and a fear of COVID-19 (1.41 [1.01–1.95]). The SEM results indicated that a fear of COVID-19, employment, trust in neighbors, and informal caregiving were indirectly associated with continued diabetes care via psychological distress. These findings suggest that a fear of COVID-19 may affect psychological distress and continued diabetes care among patients with diabetes in Japan, and that trust in neighbors and family caregiving may be related to the discontinuation of diabetes care. Therefore, because psychological factors and socioeconomic status may affect diabetes care, it is important to consider a fear of COVID-19 among patients with diabetes to maintain diabetes treatment.

## Introduction

Coronavirus disease 2019 (COVID-19) is a global public health emergency that began in 2020, resulting in numerous restrictions on the public around the world^1^. Psychological distress and socioeconomic status (SES) have been shown to be risk factors for a more severe chronic disease course^2,3^. The pandemic impinged on not only developed countries and urban contexts, but also on rural contexts^4–7^. In rural Japan, the pandemic has led to the widespread perception of COVID-19 restrictions on daily life among older adults^8^. Among patients with diabetes, psychological distress and SES are greatly influenced by a fear of COVID-19, and may eventually be associated with discontinued diabetes care^9^. While the numbers of family caregivers are increasing because of the aging of the Japanese population, informal caregivers have been shown to experience more psychological distress than the general population, even before the COVID-19 pandemic^10^. Some studies have investigated the effects of a fear of COVID-19 and psychological distress among Japanese outpatients^11,12^. To ensure continued diabetes care during the COVID-19 pandemic, many psychological effects need to be considered, even in patients without COVID-19.

To help prevent the spread of COVID-19, many countries have imposed lockdowns characterized by service closures and restrictions on outings. The Japanese government implemented a mild lockdown that was non-enforceable and non-punitive, accompanied by a declaration of a state of emergency, which attracted widespread attention^13^, on April 7, 2020 for seven prefectures (Tokyo, Kanagawa, Osaka, Saitama, Chiba, Hyogo, and Fukuoka). Tokyo, the host of the 2020 Olympics, had the highest number of infections among the 47 prefectures, and the Tokyo Metropolitan Government imposed its own restrictions on citizens^14^. The state of emergency was expanded nationwide on April 16, 2020, and lifted in a phased manner on May 14, 2020. While many countries implemented lockdowns with penalties for violations, the Japanese policy for COVID-19 was unique as the government “requested” people to refrain from going out except for emergencies and temporarily closed certain businesses without penalties for violations. This mild lockdown significantly transformed all activities, including patients’ medical examination behaviors in Japan. For example, the number of monthly train users in April 2020 decreased by 45.5% compared with April 2019^15^. Similar to the experience in other countries, the mild lockdown in Japan exerted a diverse range of effects on people’s lives, including changes in domestic circumstances (e.g., teleworking, school closures) and economic damage resulting from decreased income and job loss^16,17^. In Japan, where the mortality rate from infectious diseases was relatively low before the COVID-19 pandemic, lifestyle and daily activities associated with noninfectious diseases have had a significant impact on health^18^. Even in patients not affected by COVID-19, various aspects of diabetes care became suboptimal, leading to poor outcomes in patients with diabetes; this should also be considered as an adverse effect of the pandemic. Examining the impact of COVID-19-related emergency declarations on diabetes care in Japan is a critical issue that could indirectly affect the mortality rate during the pandemic. In a study in the United States, HbA1c testing for outpatients decreased by up to 70% during the early months of the pandemic, and this decrease in testing was associated with an increase in abnormal HbA1c results^19^.

Delays in or discontinuation of diabetes care during the pandemic represent a major problem for patients with diabetes, as these can lead to a worsening of symptoms. In Japan, it has been suggested that patients with diabetes are more likely to become severely ill because of COVID-19 infection, and it is possible that such patients may have been discouraged from seeing a doctor^9^. While fear of infection could have adverse psychological consequences in vulnerable patients with preexisting conditions, little remains known about how diabetes care has changed during the pandemic in Japan^20^. Moreover, in a previous study, the level of education attained and financial status after adjusting for psychological distress were strong predictors of mortality among patients with diabetes^21^. However, the effects of these factors on patients with diabetes in Japan during the COVID-19 pandemic remain unclear. Especially, a fear of COVID-19 can lead to reduced accessibility to diabetes care via increases in psychological distress.

**Figure 1:**
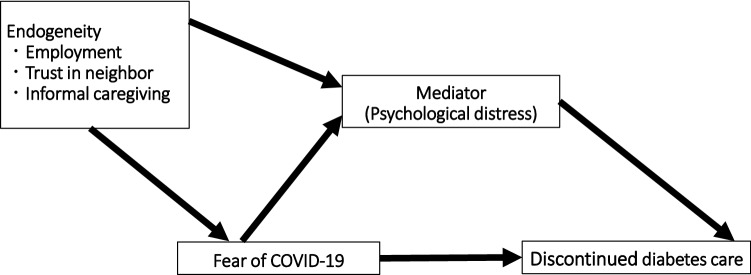
Directed acyclic graph for path analysis; each endogeneity was included in the structural equation modeling.

A directed acyclic graph of a path analysis in relation to diabetes care and a fear of COVID-19 is shown in Fig. 1. We hypothesized that a fear of COVID-19, psychological distress, and SES could affect diabetes care. Therefore, in this study, we investigated the effects of a fear of COVID-19, psychological distress, and SES on diabetes care among patients with diabetes in Japan.

## Methods

### Design, setting, and participants

The Japan COVID-19 and Society Internet Survey (JACSIS) undertook an epidemiological approach to investigate the social and individual health situations related to the COVID-19 pandemic. The Internet was estimated to be accessible to 83.4% of the Japanese population in 2020^22^. This large proportion enables researchers to use the Internet to engage numerous survey participants from a wider population range in a shorter period of time and at lower cost than conventional surveys, such as in-person interviews and mail-outs. In JACSIS, 28,000 respondents were investigated from among 224,389 qualified panelists selected through approximately 2.2 million panelists registered with a Japanese Internet survey agency (Rakuten Insight, Inc., Tokyo, Japan). These panelists are controlled and maintained by the survey agency in terms of basic information such as age and sex. The participants were recruited using a random sampling method to select a sample representative of the official demographic composition of Japan as of October 1, 2019, based on the categories of age, sex, and region of residence (i.e., all 47 prefectures). A cross-sectional design was used to detect changes in individual lifestyle and social factors before and after the COVID-19 pandemic. Web-based informed consent was obtained from all participants before they completed the online questionnaire.

Of the 28,000 respondents, 625 patients with diabetes (155 women and 470 men) were analyzed (Table 1). First, we excluded 2,518 participants who provided invalid responses. These measures to validate the quality of the data consistently were performed as described in previous studies^23–26^. Second, we excluded 23,917 participants without comorbid diabetes. Finally, we excluded participants who had not visited the hospital (n = 449) or had comorbidities (n = 490). In this survey, asthma, bronchitis, pneumonia, atopic dermatitis, otitis media, angina pectoris, myocardial infarction, stroke, chronic obstructive pulmonary disease, cancer, malignant tumors, chronic pain, depression, and mental disease (without depression) were defined as comorbidities.

**Table 1:** Characteristics of study participants with diabetes.

	Total (n = 625)	Discontinued diabetes care (n = 122 (19.5))	Continued diabetes care (n = 503 (80.5))	*P* value*
**Psychological distress**				
No (K6 score: 0–4)	498 (79.7)	85 (17.1)	413 (82.9)	0.002
Yes (K6 score: 5–24)	127 (20.3)	37 (29.1)	90 (70.9)	
**Fear of COVID-19**				
No	317 (50.7)	52 (16.4)	265 (83.6)	0.046
Yes	308 (49.3)	70 (22.7)	238 (77.3)	
**Informal caregiving**				
No	561 (89.8)	103 (18.4)	458 (81.6)	0.03
Yes	64 (10.2)	19 (29.7)	45 (70.3)	
**Sex**				
Female	155 (24.8)	39 (25.2)	116 (74.8)	0.041
Male	470 (75.2)	83 (17.7)	387 (82.3)	
**Age**				
Years	67.0 (57.0, 73.0)	64.0 (54.0, 71.0)	67.0 (58.0, 73.0)	0.012
**Educational attainment**				
High school or lower	243 (38.9)	42 (17.3)	201 (82.7)	0.261
College or higher	382 (61.1)	80 (20.9)	302 (79.1)	
**Social capital (Trust)**				
Low	174 (27.8)	32 (18.4)	142 (81.6)	0.658
High	451 (72.2)	90 (20.0)	361 (80.0)	
**Employment**				
Employer/self-employed	92 (14.7)	24 (26.1)	68 (73.9)	0.348
Regular employee	137 (21.9)	26 (19.0)	111 (81.0)	
Non-regular employee	81 (13.0)	13 (16.1)	68 (84.0)	
Unemployed	315 (50.4)	59 (18.7)	256 (81.3)	
**Income level**				
Low	204 (32.6)	45 (22.1)	159 (77.9)	0.65
Intermediate	159 (25.4)	28 (17.6)	131 (82.4)	
High	140 (22.4)	28 (20.0)	112 (80.0)	
Not answered	122 (19.5)	21 (17.2)	101 (82.8)	

Except where indicated n (%), values are median (25th, 75th percentile).

*COVID-19*: Coronavirus disease 2019.

*Wilcoxon rank sum test or χ2 test.

The study protocol conforms to the provisions of the Declaration of Helsinki. This study was reviewed and approved by the Research Ethics Committee of the Osaka International Cancer Institute (No. 20084). The data used in the present study were not deposited in a public repository because of confidentiality and restrictions imposed by the Research Ethics Committee of the Osaka International Cancer Institute. Data inquiries should be addressed to the data manager (Takahiro Tabuchi: tabuchitak@gmail.com).

### Measurements

The outcome variable was “refraining from visiting the hospital as scheduled between April and May 2020” by patients with diabetes. The outcome was obtained from a specific question: “Did you refrain from a planned hospital visit between April and May 2020?” The answer options were “yes”, “no”, and “not applicable (not planning on visiting the hospital)”. Patients with diabetes who answered “not applicable” were excluded from the analysis.

The main exposure variables were fear of COVID-19 and psychological distress. We used the Fear of COVID-19 scale (FCV-19S) to assess anxiety and fear of COVID-19. The FCV-19S has been validated in recent Japanese studies^11,27,28^. The FCV-19S is composed of seven statements, and the total score is calculated by adding up each item score (range = 7–35). The higher the score, the greater the fear of COVID-19. Total scores of 19–35 were defined as having a fear of COVID-19 (the cutoff score was defined as the median of patients with diabetes in this study). We used the 6-item Kessler Screening Scale for Psychological Distress (K6) to measure psychological distress. The total score on the K6 ranges from 0 to 24. We used a proven reliable and validated Japanese version of the K6 in this survey^29^. A K6 score ≥ 5 was used to indicate the presence of mild psychological distress^30^.

In this analysis, psychological distress was considered a mediator of a fear of COVID-19 leading to refraining from diabetes care, as described below. In a previous study, COVID-19 pandemic-associated anxiety and fear were found to be positively correlated with perceptions of vulnerability to infection^31^. We adjusted for SES as follows: informal caregiving (categorized as “yes [providing care primarily] or yes [providing care, but not primarily]” or “no [not providing]”), educational attainment (categorized as “high school or lower” or “college or higher”)^32^, employment (“employer”, “self-employed”, “regular employee”, “non-regular employee”, and “unemployed”)^33^, trust in neighbors (categorized as “high [trust or moderately trust]” or “low [not trust or moderately not trust]”)^34^, and income level (categorized by tertiles of household equivalent income in 2019 [low, < 4 million JPY; intermediate, 4–7 million JPY; high, > 7 million JPY; unknown or no answer])^35^.

### Statistical analyses

A modified Poisson model was applied to calculate risk ratios (RRs) for the association between diabetes care and mental condition (fear of COVID-19 and psychological distress) during the COVID-19 pandemic (Table 2). We designed three models to assess the effects of psychological distress and a fear of COVID-19 on diabetes care. Model 1 included only psychological distress, model 2 included a fear of COVID-19, and model 3 included both psychological distress and a fear of COVID-19. All models were adjusted for age, sex, informal caregiving, income level, employment, trust in neighbors, and educational attainment.

**Table 2:** Risk ratios for discontinued diabetes care (modified Poisson model).

	Model 1	Model 2	Model 3
	RR (95% CI)	RR (95% CI)	RR (95% CI)
**Psychological distress**			
No (K6 score: 0–4)	Reference		Reference
Yes (K6 score: 5–24)	1.44 (1.01–2.06)		1.36 (0.95–1.94)
**Fear of COVID-19**			
No		Reference	Reference
Yes		1.41 (1.01–1.95)	1.35 (0.97–1.87)
**Informal caregiving**			
No	Reference	Reference	Reference
Yes	1.52 (0.99–2.32)	1.61 (1.05–2.47)	1.53 (1.00–2.34)
**Income level**			
Low	Reference	Reference	Reference
Intermediate	0.78 (0.50–1.20)	0.77 (0.50–1.19)	0.78 (0.51–1.21)
High	0.84 (0.53–1.32)	0.82 (0.52–1.30)	0.85 (0.54–1.34)
Not answered	0.72 (0.45–1.14)	0.70 (0.44–1.12)	0.72 (0.45–1.14)
**Employment**			
Employer/self-employed	Reference	Reference	Reference
Regular employee	0.65 (0.39–1.06)	0.64 (0.39–1.06)	0.65 (0.39–1.07)
Non-regular employee	0.66 (0.36–1.21)	0.62 (0.34–1.13)	0.65 (0.35–1.18)
Unemployed	0.74 (0.47–1.18)	0.71 (0.45–1.12)	0.73 (0.47–1.15)
**Social capital (Trust)**			
Low	Reference	Reference	Reference
High	1.23 (0.85–1.78)	1.23 (0.85–1.78)	1.26 (0.87–1.82)
**Educational attainment**			
High school or lower	Reference	Reference	Reference
College or higher	1.26 (0.89–1.79)	1.30 (0.91–1.85)	1.28 (0.91–1.82)

All models were adjusted for listed variables, age and sex.

*RR* risk ratio, *CI* confidence interval, *COVID-19* coronavirus disease 2019.

To examine the statistical mediation effects, a path analysis using structural equation modeling (SEM) was performed. The SEM tested the direct effects from a fear of COVID-19 to continued diabetes care, and the indirect effects from a fear of COVID-19 to continued diabetes care via psychological distress. A fear of COVID-19 and psychological distress were modeled as continuous variables. Informal caregiving, employment, and trust in neighbors were included in the SEM as the endogeneity of both a fear of COVID-19 and psychological distress. Unstandardized profit regression coefficients with 95% confidence intervals (CIs) are reported with a root mean square error of approximation (RMSEA) ≤ 0.08^36^. Good model fit was indicated by both a goodness-of-fit index (GFI) ≥ 0.90 and an adjusted goodness-of-fit index (AGFI) ≥ 0.90.

All statistical analyses were performed using STATA 12.0 (Stata Corp, College Station, TX, USA) and JMP ver. 16.1 (SAS Institute Inc. Cary, NC, USA). This study followed the STROBE guidelines for a cross-sectional study.

## Results

Table 1 summarizes the characteristics of the study participants (N = 625). The median age of the patients with diabetes was 67.0 years, and 24.8% (n = 155) were female. In total, 19.5% (n = 122) of the patients with diabetes discontinued diabetes care during the COVID-19 pandemic. In addition, 20.3% (n = 127) had psychological distress and 49.3% (n = 308) had a fear of COVID-19. More than 70% (n = 451) of the patients with diabetes had a trusted neighbor. In terms of employment, 14.7% (n = 92) were employers or self-employed, 21.9% (n = 137) were regular employees, 13.0% (n = 81) were non-regular employees, and 50.4% (n = 315) were unemployed.

Table 2 summarizes the RRs for discontinued diabetes care with psychological distress and a fear of COVID-19 during the COVID-19 pandemic after adjusting for SES according to the modified Poisson model. In model 1, a significantly higher prevalence of discontinued diabetes care with psychological distress (RR 1.44 [1.01–2.06]) was observed after adjusting for age, sex, informal caregiving, and SES. In model 2, a significantly higher prevalence of discontinued diabetes care with a fear of COVID-19 (1.41 [95% CI = 1.01–1.95]) was found after adjusting for age, sex, informal caregiving, and SES. In model 3, slightly higher prevalences of discontinued diabetes care with both psychological distress (1.36 [0.95–1.94]) and a fear of COVID-19 (1.35 [0.97–1.87]) were found after adjusting for age, sex, informal caregiving, and SES.

**Figure 2:**
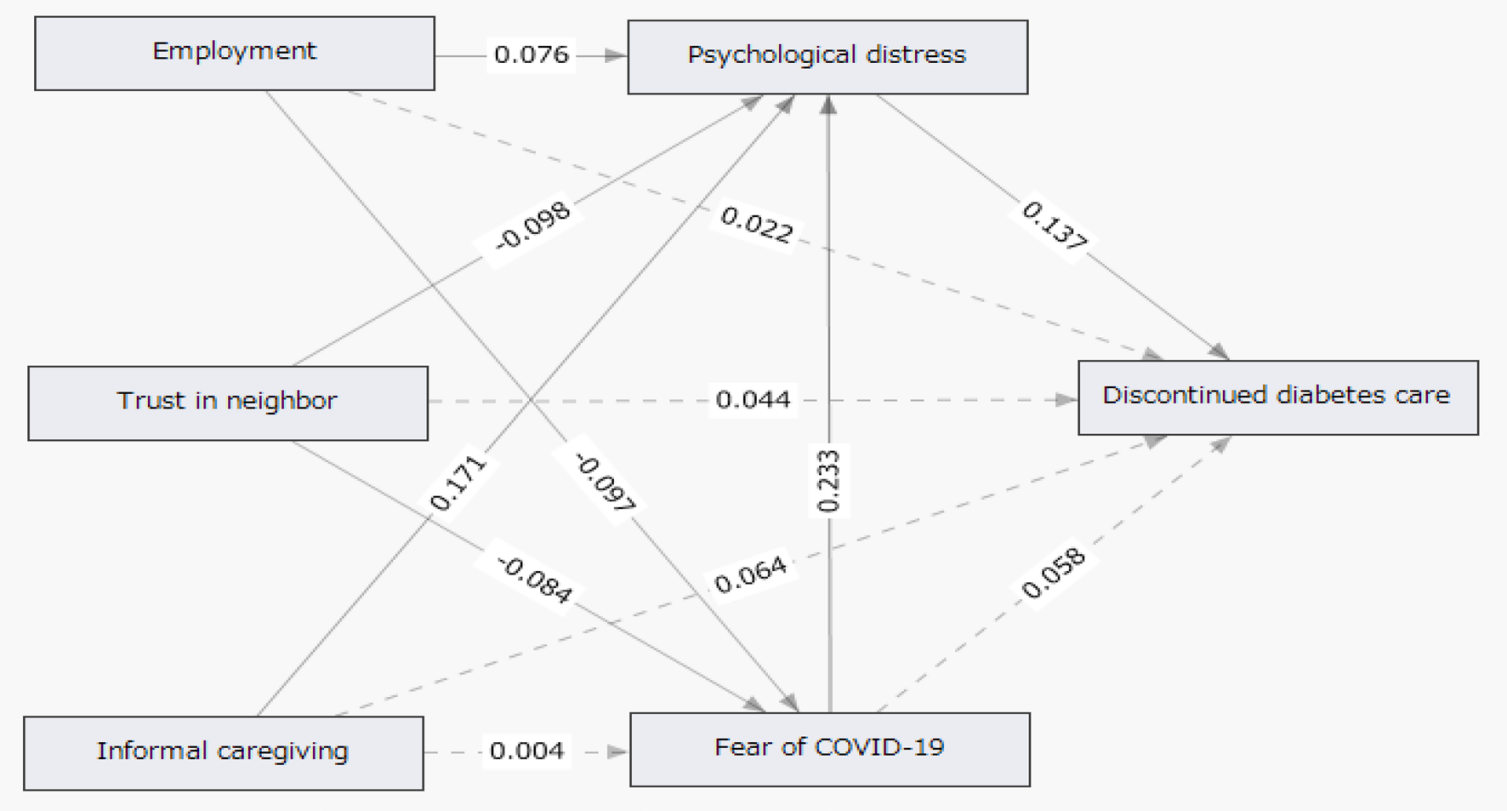
Results of structural equation modeling for the association between discontinued diabetes care and fear of COVID-19 via psychological distress. Values are standardized profit regression coefficients. Variables presented in squares are observed variables. CFI = 0.946, RMSEA = 0.051, GFI = 0.997, AGFI = 0.977. Actual lines: statistically significant (*p* < 0.05). Broken lines: not statistically significant.

Figure 2 summarizes the results of the path analysis using SEM to examine the associations among a fear of COVID-19, psychological distress, SES, and diabetes care. When a fear of COVID-19 and psychological distress were included in the path analysis model, a fear of COVID-19 via psychological distress was found to be indirectly associated with continued diabetes care. The results of the path analysis conducted with psychological distress as a mediator showed that a fear of COVID-19 was indirectly associated with discontinued diabetes care among Japanese patients with diabetes. In addition, a fear of COVID-19 was positively associated with psychological distress (path coefficient = 0.233 [0.160–0.306]), and psychological distress was positively associated with diabetes care (path coefficient = 0.137 [0.057–0.218]). A fear of COVID-19 was indirectly associated with discontinued diabetes care via psychological distress (indirect effect = 0.032 [0.009–0.067]). These findings indicated that working was significantly associated with a lower fear of COVID-19 (b =  − 0.097) and higher psychological distress (b = 0.076). Higher trust in neighbors was significantly associated with a lower fear of COVID-19 (b =  − 0.084) and lower psychological distress (b =  − 0.098) among the patients with diabetes. In addition, being a family caregiver was significantly associated with higher psychological distress (b = 0.171). Indicators of the model fit were good (CFI = 0.946, RMSEA = 0.051, GFI = 0.997, AGFI = 0.977).

## Discussion

The results of this study suggest that a fear of COVID-19 is associated with discontinued diabetes care via psychological distress. Moreover, a fear of COVID-19 and psychological distress tend to be affected by employment, trust in neighbors, and family caregiving. While patients with underlying illnesses may be more likely to become severely ill when infected with COVID-19 via their family, informal caregivers may be more likely to discontinue diabetes care and avoid visiting the hospital^37^.

To our knowledge, this is the first study to examine the association between a fear of COVID-19 leading to refraining from care among patients with diabetes in Japan. The results of the path analysis conducted with psychological distress as a mediator in the SEM showed that a fear of COVID-19 was indirectly associated with discontinued diabetes care among patients with diabetes in Japan. Patients with diabetes and poor glycemic control are at a higher risk of a severe case when infected with COVID-19^38^. Regarding the association between a fear of COVID-19 and discontinued diabetes care, patients with poor glycemic control are also at higher risk of a severe case when infected with COVID-19^39^. Thus, patients with diabetes may have chosen to avoid outpatient treatment to help prevent COVID-19 infection rather than continued diabetes care. In addition, the psychological distress derived from a fear of COVID-19 may have reduced the motivation for diabetes care among these patients. Previous studies have suggested that psychological distress may be a barrier to diabetes care, which is consistent with our results^38,40^.

The findings of this study suggest that trust in neighbors affects the fear of COVID-19 and psychological distress among patients with diabetes. A Chinese study conducted before the COVID-19 pandemic reported that more attention should be paid to the mental health of critically ill patients and formal social, community, and governmental support, particularly in rural China^41^. The results of that study were consistent with the findings of the present study, in that trust in neighbors may have been associated with diabetes care during the COVID-19 pandemic. Medical treatment-based information and communication technology were more noticeable during the COVID-19 pandemic because of infection control and rural medicine. Telemedicine may be promising for continued diabetes care while reducing the risk of COVID-19 infection, given that the number of reimbursement codes for telemedicine use is still small in Japan (accounting for only 0.02% of all outpatient care services)^42^. Barone et al. reported that to protect communities, health systems, and the global economy, citizens should value the knowledge and experience gained from past outbreaks and the lessons learned from the COVID-19 pandemic, allowing them to take immediate action to protect the most vulnerable groups^43^. In addition, a recent study reported associations according to the number of risk factors between incident dementia, cognitive performance, and brain abnormalities among individuals with type 2 diabetes when compared with control subjects without diabetes^44^. While SES among patients with diabetes may be obscured or change rapidly because of the prolonged COVID-19 pandemic, attention should be paid to the patients’ background as they continue to receive diabetes care.

Regarding the impact of these findings on long-term diabetes care in relation to informal caregiving, there were two possible effects: (1) patients with diabetes could not undergo treatment because of insufficient time, and (2) patients had to cancel or postpone treatment because of infection control for their family (especially caregivers at home). During the COVID-19 pandemic, disruptions in the formal care system and medical care system have increased the need for more intense informal care at home^45^. The predicted effect of trust in neighbors on diabetes care is that people with higher trust in neighbors will be more likely to have access to accurate medical information about diabetes treatment. The findings of the present study suggest that a fear of COVID-19 is associated with discontinued diabetes care, and that trust in neighbors and family caregiving buffer discontinued diabetes care.

The main strengths of this study are its assessment of various individual-level factors and the large-scale sample covering all 47 prefectures in Japan during the COVID-19 pandemic. On the other hand, this study had several limitations. First, we cannot clarify the direction of causality because this was a cross-sectional study. However, we assumed a plausible causal direction from a fear of COVID-19 leading to refraining from diabetes care via psychological distress and estimated the effect based on this assumption. Second, the data used in this study were obtained from an Internet survey, and the response rate was relatively low (12.5%). We adjusted as much as possible to account for possible biases in the collected sample using an external, nationally representative sample. Third, our findings may not be generalizable to the population with limited access/literacy to the Internet because our study sample was collected through an Internet survey. However, most Japanese people have been able to access the Internet during the pandemic, so we concluded that this would not have substantially affected the results of the present survey. Fourth, the study participants included many older adults, and their SES may differ from that of younger cohorts. On this point, caution should be exercised in generalizing the results. Finally, there was no medical diagnosis of diabetes, so we could not use an accurate measurement of diabetes intensity or care plan. Due to social distancing requirements, reduced mobility, and meeting restrictions, it was not possible to include certain measurements, which could have provided more relevant information regarding the risk factors of discontinued diabetes care. As the lockdown owing to the COVID-19 pandemic began around March 2020, there may have been a time lag of up to 5 months to when diabetes care was not maintained. Although it has been difficult for patients with diabetes to be observed and treated during the COVID-19 pandemic, the results of this study could provide valuable data in regard to diabetes care, psychological factors, and SES.

## Conclusions

These findings suggest that a fear of COVID-19 may affect psychological distress and continued care among patients with diabetes in Japan, and that trust in neighbors and family caregiving may be related to discontinued diabetes care. As psychological factors and SES may affect diabetes care, it is important to consider a fear of COVID-19 among patients with diabetes to maintain diabetes treatment.
